# PREDICTORS OF FAMILY FUNCTIONING IN CUSTODIAL GRANDFAMILIES: A SYSTEMIC PERSPECTIVE

**DOI:** 10.1093/geroni/igad104.3068

**Published:** 2023-12-21

**Authors:** Khushbu Patel, Deneisha Scott-Poe, Megan Dolbin-MacNab

**Affiliations:** Virginia Tech, Blacksburg, Virginia, United States; Virginia Tech, Blacksburg, Virginia, United States; Virginia Tech, Blacksburg, Virginia, United States

## Abstract

According to the U.S. Census, there are approximately 2.5 million grandparents raising grandchildren. Previous research has examined factors associated with grandparent and grandchild well-being; yet, few studies have considered the functioning of the grandfamily unit. Guided by family systems and attachment theories, the purpose of this study was to examine individual and relational predictors of grandfamily functioning, as reported by 95 custodial grandmothers raising adolescent (ages 12 to 18) grandchildren. Survey data were collected in-person or over the telephone from custodial grandmothers residing in the Southern U.S. To analyze the data, we conducted a hierarchical multiple regression. The first model included grandchild gender, the second model added grandmothers’ parenting stress and attachment, and the final model further added grandmothers’ reports of grandchildren’s attachment, emotion regulation, and internalizing and externalizing behavior problems. The first model was not significant, but the second model was significant [F (3, 91) = 8.85, p < .001; R2 Δ = .22, p < .001], and explained 23% of the variance in grandfamily functioning, with less grandmother parenting stress predicting better grandfamily functioning. More secure grandmother attachment approached significance. The final model accounted for 34% of the variance in grandfamily functioning [F (7, 87) = 6.35, p < .001; R2 Δ = .11, p = .008], with better grandfamily functioning being predicted by less grandmother parenting stress and better grandchild emotion regulation. More secure grandmother attachment and greater grandchild internalizing problems emerged as trends. Implications for future research and systemic practice with grandfamilies will be discussed.

